# Identifying cell types from single-cell data based on similarities and dissimilarities between cells

**DOI:** 10.1186/s12859-020-03873-z

**Published:** 2021-05-18

**Authors:** Yuanyuan Li, Ping Luo, Yi Lu, Fang-Xiang Wu

**Affiliations:** 1 School of Mathematics and Physics, Wuhan Institute of Technology, No.206, Guanggu 1st road, Wuhan, 430205 Hubei China; 2 Division of Biomedical Engineering, University of Saskatchewan, 57 Campus Drive, Saskatoon, SK S7N 5A9 Canada; 3 Department of Mechanical Engineering, University of Saskatchewan, 57 Campus Drive, Saskatoon, SK S7N 5A9 Canada; 4 Department of Computer Science, University of Saskatchewan, 57 Campus Drive, Saskatoon, SK S7N 5A9 Canada

**Keywords:** Single-cell data, Spectral clustering, Similarity/dissimilarity matrix, Cell types identification

## Abstract

**Background:**

With the development of the technology of single-cell sequence, revealing homogeneity and heterogeneity between cells has become a new area of computational systems biology research. However, the clustering of cell types becomes more complex with the mutual penetration between different types of cells and the instability of gene expression. One way of overcoming this problem is to group similar, related single cells together by the means of various clustering analysis methods. Although some methods such as spectral clustering can do well in the identification of cell types, they only consider the similarities between cells and ignore the influence of dissimilarities on clustering results. This methodology may limit the performance of most of the conventional clustering algorithms for the identification of clusters, it needs to develop special methods for high-dimensional sparse categorical data.

**Results:**

Inspired by the phenomenon that same type cells have similar gene expression patterns, but different types of cells evoke dissimilar gene expression patterns, we improve the existing spectral clustering method for clustering single-cell data that is based on both similarities and dissimilarities between cells. The method first measures the similarity/dissimilarity among cells, then constructs the incidence matrix by fusing similarity matrix with dissimilarity matrix, and, finally, uses the eigenvalues of the incidence matrix to perform dimensionality reduction and employs the K-means algorithm in the low dimensional space to achieve clustering. The proposed improved spectral clustering method is compared with the conventional spectral clustering method in recognizing cell types on several real single-cell RNA-seq datasets.

**Conclusions:**

In summary, we show that adding intercellular dissimilarity can effectively improve accuracy and achieve robustness and that improved spectral clustering method outperforms the traditional spectral clustering method in grouping cells.

## Background

In recent years the development of single-cell sequencing technologies opens a new point of view on a series of complex biological phenomena at the single-cell level [1]. Rich datasets produced with these technologies can be utilized to investigate differences in gene expression between individual cells, characterize cell types, and study heterogeneity in cell line [2]. Nevertheless, different types of cells are often infiltrated into each other in the traditional biological experiments [3]. An effective way of solving this problem would be to group individual cells by using the method of clustering so that cells within the same cluster establish extremely similar patterns of gene expression.

The process of grouping cells based on single-cell data is an unsupervised clustering problem, and a collection of computational methods have been presented to sort out this problem such as hierarchical analysis [4], K-means [5], principal component analysis (PCA) [6] and spectral clustering [7]. However, potential technical and biological issues bring great challenges such as much noise, many missing values, high gene expression variability and so on [8]. In addition, the number of genes assayed in scRNA-seq is much larger than the number of cells for classification, which may lead to the distances between cells become similar. Accordingly, most of traditional clustering algorithms lose their action in partitioning the cells into well-separated groups.

Many people have worked hard to circumvent these problems in recent years, they have tried their best to define cell types on the basis of single-cell gene expression patterns. For example, Buettner et al.  [9] presented a single-cell latent variable model to identify otherwise undetectable subpopulations of cells. Xu and Su used the conception of shared nearest neighbor and proposed a novel algorithm named shared nearest neighbor (SNN)-Cliq that groups cells, which could generate desirable solutions with high accuracy and sensitivity [10]. Höfer and Shao adapted Nonnegative Matrix Factorization (NMF) [11, 12] to study the problem of the unsupervised learning of cell subtypes from single-cell gene expression data [13]. Kiselev et al. [14] put forward single-cell consensus clustering (SC3), which combined all the different clustering outcomes into a consensus matrix and determined the final results by complete-linkage hierarchical clustering of the consensus matrix. Lin et al. [15] incorporated prior biological knowledge to test various neural networks architectures and used these to obtain a reduced dimension representation of the single-cell expression data for identifying a unique group of cells. Gao et al. [16] adopted a likelihood-based strategy using the two-state model of the stochastic gene transcription process and developed Clustering And Lineage Inference in Single Cell Transcriptional Analysis (CALISTA) for clustering and lineage inference analysis. Zheng et al. [17] drew inspiration from the self-expression of the cells with the same group, imposed the non-negative and low rank structure on the similarity matrix, and then proposed a SinNLRR method for scRNA-seq cell type detection. Zhu et al. [18] explored a method by combining structure entropy and k nearest neighbor to identify cell subpopulations in scRNA-seq data. Jiang et al. [19] proposed a new cell similarity measure based on cell-pair differentiability correlation and further developed a variance analysis based clustering algorithm that can identify cell types accurately. For identifying cell subtypes, most of these approaches do reasonably well for some situations by employing feature selection or dimensionality reduction to reduce the noise of original data and speed up the calculation processes [20].

Spectral clustering (SC), as one of the most popular modern clustering algorithms, uses the first k eigenvectors of the Laplacian matrix derived from the similarity matrix to carry out dimensionality reduction for clustering. SC is very easy to implement and can be realized efficiently by using standard linear algebra methods [21]. Generally speaking, there are three methods for constructing a similarity matrix: $\epsilon$-neighborhood, k-nearest neighbor, or fully connected. All methods are based on using distance measurement by several different choices available, including Euclidean distance, Pearson’s correlation, Spearman’s correlation, Gaussian similarity function and so on. In general, the performance of clustering is quite sensitive to the choice of similarity measurement. Lately, there are several computational analysis methods available to improve the clustering effect of SC. For instance, Lu et al. [22] proposed a convex Sparse Spectral Clustering (SSC) model which extended the traditional spectral clustering method with a sparse regularization and proposed the Pairwise Sparse Spectral Clustering (PSSC) method which seeks to improve the clustering performance by leveraging the multi-view information. Wang et al. [23] combined multiple kernels to fit the structure of the data best and employed a rank constraint in the learned cell-to-cell similarity and graph diffusion in order to perform dimension reduction, clustering, and visualization. Park and Zhao utilized multiple doubly stochastic similarity matrices to learn a similarity matrix and imposed a sparse structure on the target matrix followed by shrinking pairwise differences of the rows in the target matrix to extend spectral clustering algorithm [24].

Although these methods can get promising effect in identifying cell types, they only consider the impact of the positive similarities between cells on the clustering result and not consider the impact of negative similarities. That is to say, only the similarities are considered, but the dissimilarities are overlooked. This methodology may have limitation on the effectiveness of those clustering algorithms based on spectral analysis for grouping cells that belong to the same cell types. However, the intuitive goal of SC is to divide the data points (representing single cells) into several groups such that points in the same group are similar and points in different groups are dissimilar to each other [21]. Hence, dissimilarities between single cells should not be ignored. In this study, we build a suitable incidence matrix considering similarities as well as dissimilarities between cells meanwhile and improve spectral clustering method for partition cells. In the process of our improved algorithm, we adopt the dissimilarity matrix to stress the dissimilarities between the natural groupings, and a parameter is adjusted to balance the similarity matrix and dissimilarity matrix.

To investigate the performance of the improved method, we first apply it in breast cancer data to distinguish tumor cells, stromal cells, and immune cells and compare the results with the conventional SC. Then we apply it to other four scRNA-seq datasets which are characterized as highly confident in the cell labels. Our result shows that taking into account similarities as well as dissimilarities increase performance. Moreover, the clustering results indicate that the improved method gets higher accuracy and strong robustness in identifying cell subpopulations.

## Results

We applied the improved spectral clustering (ISC) method to several published single cell datasets. The results were compared with conventional spectral clustering by Purity, Rand Index (RI), Adjusted Rand Index (ARI) and Normalized Mutual Information (NMI).

### Breast cancer data

The first biological dataset we tested had RNA-seq data of 549 single cells. After the filtering steps as described in the method session, 34 single cells with low sequencing quality were discarded. Among the remaining 515 single cells, it has been testified that there were 317 epithelial breast cancer cells, 175 tumor-associated immune cells and 23 non-carcinoma stromal cells, that can be considered gold standards. 11986 genes were selected by strict quality control and the gene normalizations were implemented before they were capable of clustering the cells into distinct groups.

**Fig. 1:**
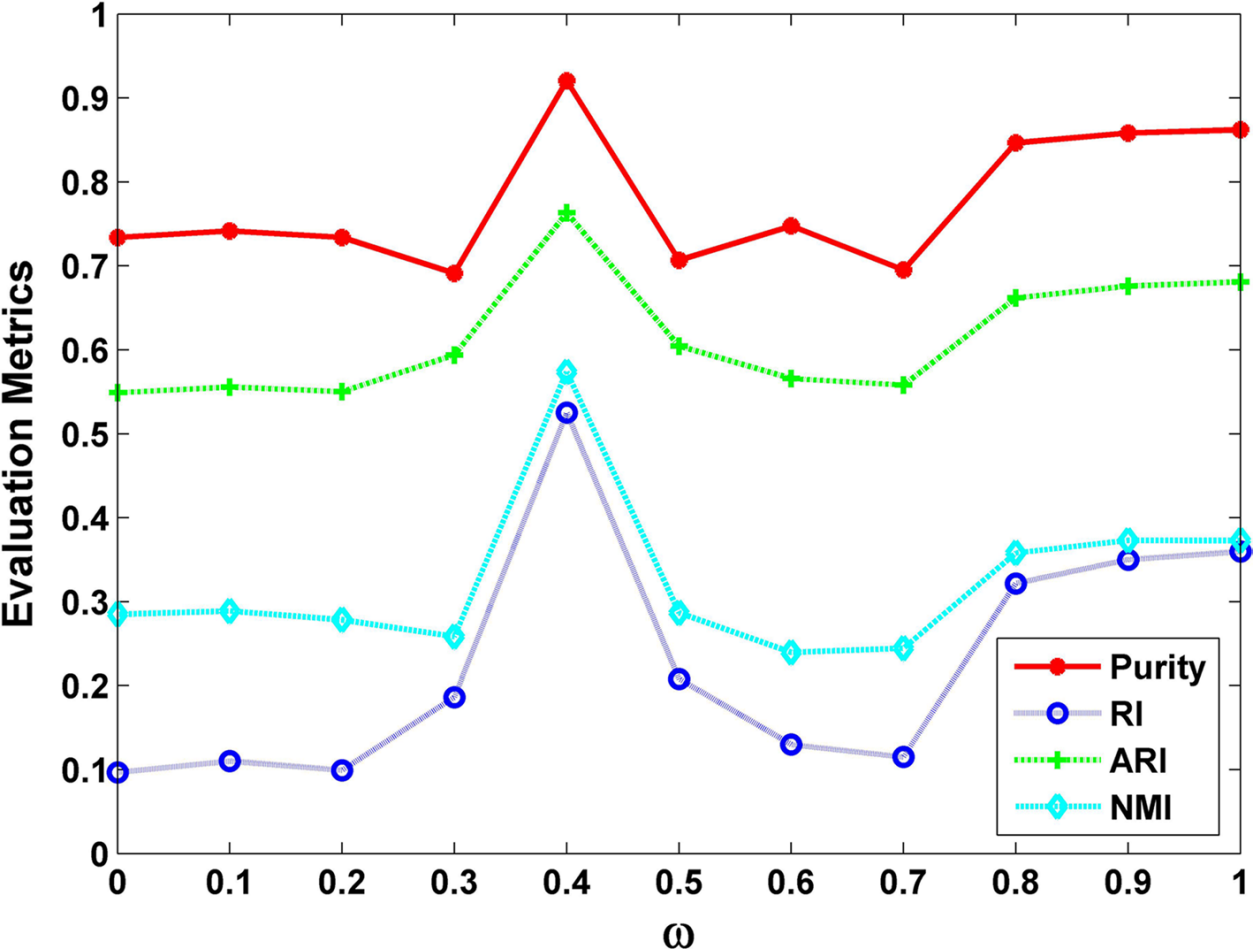
The performance of the improved spectral clustering with the variation of parameter $\omega$ when the values of *h* and *q* are fixed to 80. Purity, RI, ARI and NMI all reach their maximum values when $\omega$ is equal to 0.4

The parameter $\omega$ is provided to trade off the weight between similarity and dissimilarity on the incidence matrix. The value of $\omega$ has to be between 0 and 1. As the value of $\omega$ gets smaller, the more emphasis is put on the similarity inside a cluster, especially, when $\omega$ equals zero, the improved spectral clustering is the conventional spectral clustering. The closer that the value of $\omega$ is to 1, the more attention is paid to the dissimilarity between clusters. When *h* and *q* are fixed to 80, the performance of improved spectral clustering with the change of parameter $\omega$ is shown in Fig. 1. As can be seen from Fig. 1, with the parameter $\omega$ grows, Purity, RI, ARI and NMI values all maintain steady in the beginning, then increase drastically and all reach their maximum values when $\omega$ is equal to 0.4, and then these indices fall back quickly, lastly they rise to become stable. It can be obtained that the clustering results of improved spectral clustering (when $\omega$ is equal to 0.4) are better than the performance of conventional spectral clustering (when $\omega$ is equal to 0). This demonstrates that when using spectral clustering algorithm taking both the similarity within the cluster and the dissimilarity between clusters into account can’t be worse than only considering similarity within the cluster.

In the implementation process of the improved spectral clustering, there are other two required parameters, *h* and *q*, which represent the width of similar neighborhoods and dissimilar neighborhoods, respectively. In this study, the effects of each parameter to clustering results are discussed. If the number of cells is *ns* and the number of cell types is *nt*, we first round *ns* up to the nearest hundreds and divide it by 100 as step-size *ss*, then we increase the *h* from *ss* to $0.5\times ns/nt$ with interval *ss* for studying the influence of the parameter *h*. For example, there are 3 cell types of 515 cells in breast cancer dataset, we consider $h\in \{5,10,15,20,\ldots ,80,85\}$, and when the value of *h* is given, *q* is set equal to *h*, or equal to *h*/2. Thus, the incidence matrix can be obtained by 32 different parameter combinations. The best performance of improved spectral clustering with different parameter combinations of *h* and *q* is listed in Table 1. When only the similarity is considered, $\omega$ is set to zero, the improved spectral clustering is the conventional spectral clustering. When in consideration of similarity and dissimilarity, $\omega$ is set to a non-zero value. It can be drawn from Table 1 that improved spectral clustering performs better with various combinations of *h* and *q* settings in breast cancer dataset. Although $\omega$ is different when improved spectral clustering is in the best performance according to different combinations of *h* and *q*, the results show the better robustness and our improved algorithm is also insensitive to the values of parameter *h* and *q*.

**Table 1:** The best performance of improved spectral clustering with different parameter combinations of *h* and *q*

*h*	*q*	*ω*	Purity	RI	ARI	NMI
5	0	0	0.6155	0.4668	− 0.0627	0.1506
5	2	0.5	0.8446	0.6938	0.3861	0.3934
5	5	0.1	0.9223	0.7545	0.5076	0.5556
10	0	0	0.6155	0.467	− 0.0626	0.1593
10	5	0.4	0.8582	0.6987	0.3958	0.3993
10	10	0.4	0.8718	0.7098	0.4181	0.4203
15	0	0	0.6155	0.4669	− 0.062	0.1582
15	7	0.2	*0*.*9281*	0.7588	0.5161	*0*.*5784*
15	15	0.2	0.9262	0.7449	0.4883	0.5565
20	0	0	0.6155	0.4682	− 0.0607	0.1686
20	10	0.7	0.866	0.7023	0.4031	0.3968
20	20	0.9	0.8718	0.7091	0.4168	0.4045
25	0	0	0.6155	0.4873	− 0.0239	0.2107
25	12	0.1	0.864	0.678	0.3545	0.422
25	25	0.3	0.9281	0.7463	0.4912	0.5621
30	0	0	0.6252	0.4925	− 0.0139	0.202
30	15	0.3	0.9262	0.7457	0.4898	0.5656
30	30	0.3	0.9242	0.7587	0.516	0.5574
35	0	0	0.6291	0.4941	− 0.0109	0.2049
35	17	1	0.866	0.706	0.4105	0.3899
35	35	0.3	0.9262	0.7623	0.5232	0.566
40	0	0	0.631	0.4926	− 0.0137	0.212
40	20	0.4	0.9242	0.7442	0.487	0.5505
40	40	0.4	0.92233	0.7437	0.4859	0.5508
45	0	0	0.631	0.4926	− 0.0137	0.212
45	22	0.4	0.9184	0.7345	0.4674	0.5417
45	45	0.4	0.9203	0.7495	0.4975	0.5513
50	0	0	0.6951	0.523	0.0455	0.256
50	25	0.1	0.9242	0.7526	0.5037	0.5498
50	50	1	0.8699	0.6973	0.3928	0.3924
55	0	0	0.7029	0.5275	0.0544	0.2627
55	27	0.9	0.8737	0.7185	0.4355	0.4164
55	55	0.1	0.9203	0.7479	0.4944	0.5393
60	0	0	0.7067	0.5299	0.0592	0.2662
60	30	0.4	0.8834	0.7169	0.432	0.4735
60	60	1	0.8796	0.7015	0.4014	0.4082
65	0	0	0.7165	0.5363	0.0718	0.2751
65	32	0.9	0.8757	0.7153	0.4292	0.4188
65	65	1	0.8776	0.6998	0.398	0.4044
70	0	0	0.7242	0.5415	0.0822	0.2823
70	35	0.4	0.9126	0.7498	0.4982	0.5474
70	70	1	0.8776	0.6993	0.3969	0.4041
75	0	0	0.73	0.5457	0.0906	0.2879
75	37	0.6	0.8854	0.7103	0.419	0.4286
75	75	0.4	0.9203	0.7607	0.5199	0.5715
80	0	0	0.7339	0.5488	0.0967	0.2849
80	40	0.9	0.8815	0.713	0.4245	0.4229
80	80	0.4	0.9203	*0*.*7633*	*0*.*5252*	0.5735
85	0	0	0.7417	0.5544	0.1076	0.2925
85	42	0.6	0.8893	0.7065	0.4113	0.4311
85	85	0.4	0.9184	0.7603	0.5192	0.5552

As the value of *h* increases, the conventional spectral clustering is getting better and better. when $h=85$, $q=0$, the conventional spectral clustering has the best performance, the Purity, RI, ARI and NMI values are 0.7417, 0.5544, 0.1077 and 0.2915, respectively. But no matter what the values of *h* and *q* are, improved spectral clustering shows stable performance. When $h=15$, $q=7$ and $\omega =0.2$, the improved spectral clustering gains the best clustering results in terms of Purity and NMI, which are 0.9281 and 0.5784, respectively. When $h=80$, $q=80$ and $\omega =0.4$, the improved spectral clustering performs best in terms of RI and ARI, which are 0.7633 and 0.5252, respectively. Although, the clustering results of improved spectral clustering are pretty good, the ARI value and NMI value are not so satisfactory, they are still less than 0.6. Maybe it is because, among three types of cells isolated from individual tumor tissues, tumor cells have distinct chromosomal expression patterns, recapitulating tumor-specific copy number variations while immune cells and stromal cells have no apparent copy number variation patterns [3]. The separation of the latter two types of cells become a little difficult by the clustering method based on gene expression pattern.

**Fig. 2:**
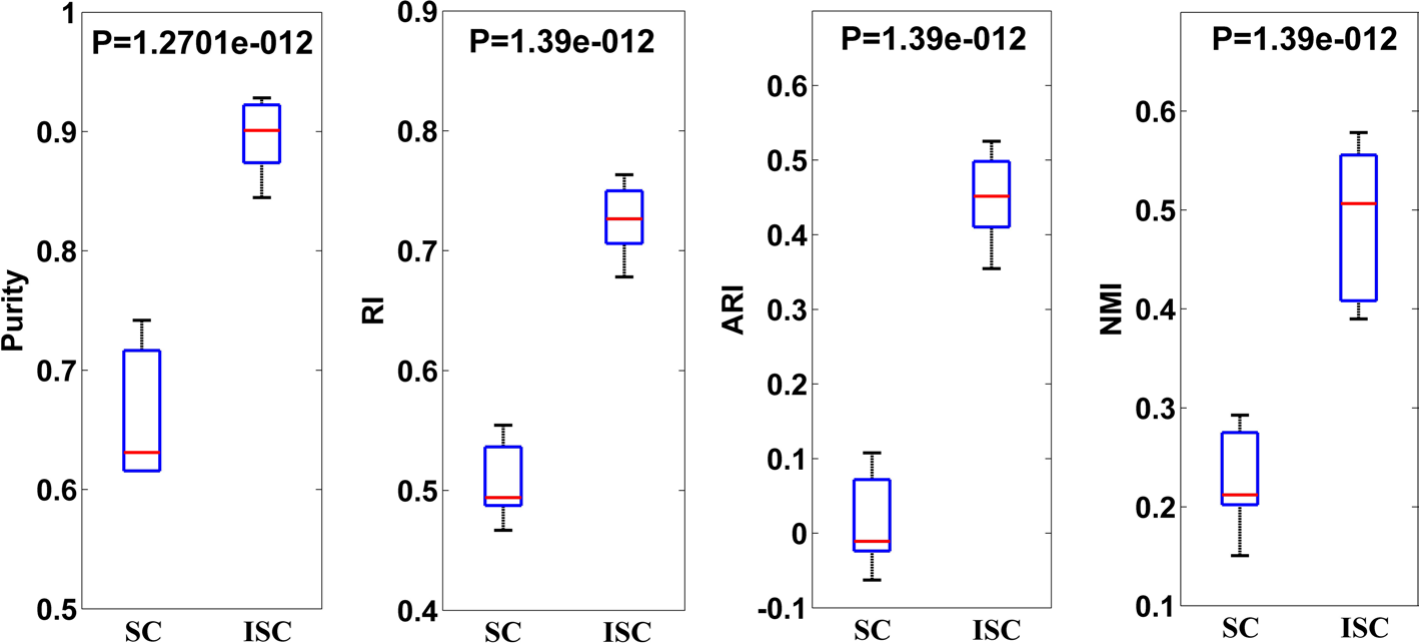
Comparison of the best performance between spectral clustering and improved spectral clustering using the same values for parameters *h* and *q*. SC denotes conventional spectral clustering and ISC denotes improved spectral clustering. P-values are from a one-tailed Wilcoxon rank sum test. Evaluation metrics in the ISC exhibit significantly higher values than those in the SC

Moreover, to determine whether the improved spectral clustering is significantly better than the conventional spectral clustering, we use the non-parametric one-tailed Wilcoxon rank sum test. We calculate the P-value of the test, as shown in Fig. 2, and take it as the significant levels of difference between the improved spectral clustering and the conventional spectral clustering. To test for a difference in the evaluation metrics of improved spectral clustering and conventional spectral clustering, we use the following procedures. We first calculate the evaluation metrics of improved spectral clustering and conventional spectral clustering with various $\omega$ value for given values of *h* and *q* and record the best performance of improved spectral clustering and conventional spectral clustering. This process was repeated when *h* and *q* are changed at the same time. The significance level of the tests is then calculated by the proportion of the evaluation metrics of the conventional spectral clustering that exceeds the evaluation metrics of the improved spectral clustering. Calculation and comparisons show that the evaluation metrics of improved spectral clustering is significantly greater than those of conventional spectral clustering and there is remarkable differences between them.

### Other real data

we then compare our proposed improved spectral clustering with the conventional spectral clustering on other four single-cell RNA sequence datasets featuring high-confidence cell labels. These datasets are derived from different single-cell RNA-seq techniques and are collected from human or mouse. Some cells involve in different biological process, some are original from different tissues, and some are generated from different lines [25–28]. All the original expressions have been pre-processed in previous study [23, 24]. Dendritic cells (DCs) dataset consists of 251 cells at three different progenitor stages and 11834 genes which pass the gene filter step. A mixture of diverse single cells (MCs) dataset consists of 249 single cells were captured from a mixture of 11 cell populations. After initial filtering steps similar to DCs dataset above, 14805 genes remained for further analysis. Embryonic stem cells (ESCs) dataset consists of 704 cells grown in three different conditions. There are 10685 genes which passed the quality control. Neuronal cells (NCs) dataset consists of 622 individual cells, after quality control analysis on each individual cell, 17772 genes were selected for downstream analysis. we use the true cluster number to obtain the clustering results.

According the way to determine the value of *h* in the breast cancer data. The value of *h* is from $\{3,6,9,12,\ldots ,39,42\}$ in DCs dataset, is from $\{2,4,6,8,\ldots ,10,12\}$ in MCs dataset, is from $\{7,14,21,28,\ldots ,112,119\}$ in ESCs dataset and is from $\{6,12,18,24,\ldots ,72,78\}$ in NCs. when *h* is given a fixed value, *q* is set equal to *h*, or equal to *h*/2. Then improved spectral clustering method with different combinations of parameters are applied to clustering cells in these datasets. Table 2 shows the best performance of traditional spectral clustering ($\omega =0$) and improved spectral clustering ($\omega \ne 0$). From the four index values given in the Table 2, it can be seen that the improved spectral clustering is a notch above the conventional spectral clustering. By improved spectral clustering, Purity, RI, ARI and NMI are all increased in some degree, the biggest rise with a 23.3$\%$ increase. Furthermore, we can see that in MCs dataset, although the clustering results of conventional spectral clustering have been proved to be satisfactory, improved spectral clustering can get better results. Although ARI and NMI are increasing in DCs dataset, they are still low, perhaps this is because although progenitor populations retained expression of surface markers at the protein level associated with the respective specific progenitor stages, individual cells had already shifted transcriptionally toward the next step in differentiation, there existed a significant overlap in gene-expression profiles among the development of dendritic cell subsets [25].

**Table 2:** The best performance of spectral clustering and improved spectral clustering on other real data

Datasets	*h*	*q*	*ω*	Purity	RI	ARI	NMI
DCs	39	0	0	0.6653	0.6611	0.3151	0.3706
	36	36	0.2	0.6813	0.7108	0.38	0.4451
MCs	12	0	0	0.9598	0.9852	0.9208	0.955
	12	12	0.1	0.9638	0.9889	0.9413	0.9604
ESCs	63	0	0	0.8807	0.8741	0.7207	0.7701
	14	14	0.3	0.9318	0.9212	0.8275	0.7942
NCs	72	0	0	0.7894	0.8233	0.5495	0.6421
	30	30	0.3	0.8328	0.8719	0.6731	0.6792

## Discussion

Large volume of single cell data have emerged in response to the progress of next-generation sequencing technology, how to take full advantage of these rich data is very important. One of the most powerful applications of single-cell data is to define cell types by clustering analyse on the basis of gene expression patterns. The clustering qualities have an effect on the outcome of downstream analysis. Up to now, many clustering algorithms for identifying subtypes of cells have been proposed.

Owing to the high dimensionality of the single-cell data, the gaps among the distances between cells narrow. Thus, it is unreliable to define cell types on the basis of these high-dimensional data directly. Effective dimensionality reduction could make the measure of the distance between cells more accurate in cells clustering. For example, spectral clustering projects data into a lower-dimensional space based on the eigenvectors corresponding to the *k* smallest eigenvalues of the Laplace matrix, and Laplace matrix is deduced according to the incidence matrix. However, the general method for constructing the incidence matrix only attaches importance to similarities between cells and overlooks the dissimilarities between cells. The dissimilarities between cells contain the discrepancy in expression pattern between different cell types and have very influential consequences in identifying clusters. We expect that imposing the dissimilarities between cells can help to achieve better clustering results.

In this study, the conventional spectral clustering method has been improved for clustering single cells by the combination of similarities and dissimilarities between cells. Furthermore, we apply this improved method to five published single-cell datasets including cells from different tissues, stages, cell lines and so on. The results show that it performs better than conventional spectral clustering based on several metrics. Through the integration of similarities and dissimilarities, the classification accuracy is improved. The performance of the proposed method with various parameter combinations also shows the better robustness of the improved method.

Although improved spectral clustering makes some progress in identifying cell types, the ability to detect cell types still could be developed the most. Several problems are really challenges, which include what measurements are used to reflect the distance between cells, how to reasonably measure the similarities and dissimilarities, how many similar cells and dissimilar cells are to choose for constructing similarity matrix and dissimilarity matrix and how to balance similarity matrix and dissimilarity matrix to construct incidence matrix. The answers to these questions depend on specific data and solving these problems will require data-driven approaches. In addition, the prediction of the number of clusters is a challenge. In the future, it would be interesting to develop a more effective clustering method by integrating improved spectral clustering and other computational analysis methods.

## Conclusion

In this study, we have improved conventional spectral clustering algorithm for separating single cells into distinct groups by incorporating dissimilarities between cells with similarities. We have shown that its performance is superior to conventional spectral clustering method on several published single cell datasets.

## Methods

### Data sources

In this study, we used five published single-cell datasets. At first, we put emphasis on the analysis of primary breast cancer cells (BCCs). The original single-cell RNA sequencing was downloaded from the NCBI GEO database under the accession code GSE75688 [3]. Eleven primary tumor specimens and two metastatic lymph nodes were collected and processed for single-cell RNA sequencing. In total, 549 single-cell cDNAs were subjected to RNA sequencing.

Then, we directly applied the improved algorithm to other processed single-cell gene expression datasets from previously published papers [23, 24]. DCs arise from a cascade of progenitors that gradually differentiate in the bone marrow [25]. Schlitzer et al. used mRNA sequencings of 251 dendritic cell progenitors to investigate the transcriptomic relationships. Those dendritic cell progenitors had been in one of the following three cellular states: macrophage dendritic cell progenitor, common dendritic cell progenitor, and pre-dendritic cell. Pollen et al. [26] made an unbiased analysis and comparison of 249 MCs with greater than 500,000 reads from 11 populations by microfluidic single-cell capture and low-coverage sequencing of many cells. Kolodziejczyk et al. [27] collected 704 single-cell transcriptomes of ESCs cultured in three different conditions: serum, 2i, and the alternative ground state a2i and studied on how different culture conditions influence pluripotent states of ESCs. Usoskin et al. [28] used comprehensive transcriptome analysis of 622 single mouse NCs for identification of four neuronal groups, which reveals the diversity and complexity of primary sensory system underlying somatic sensation. The basic information for the above-mentioned single-cell datasets is listed in Table 3.

**Table 3:** Basic information for five single-cell datasets

Datasets	Number of cells	Number of cell types	Reference
BCCs	549	3	[3]
DCs	251	3	[25]
MCs	249	11	[26]
ESCs	704	3	[27]
NCs	622	4	[28]

### Data preprocessing

**Fig. 3:**
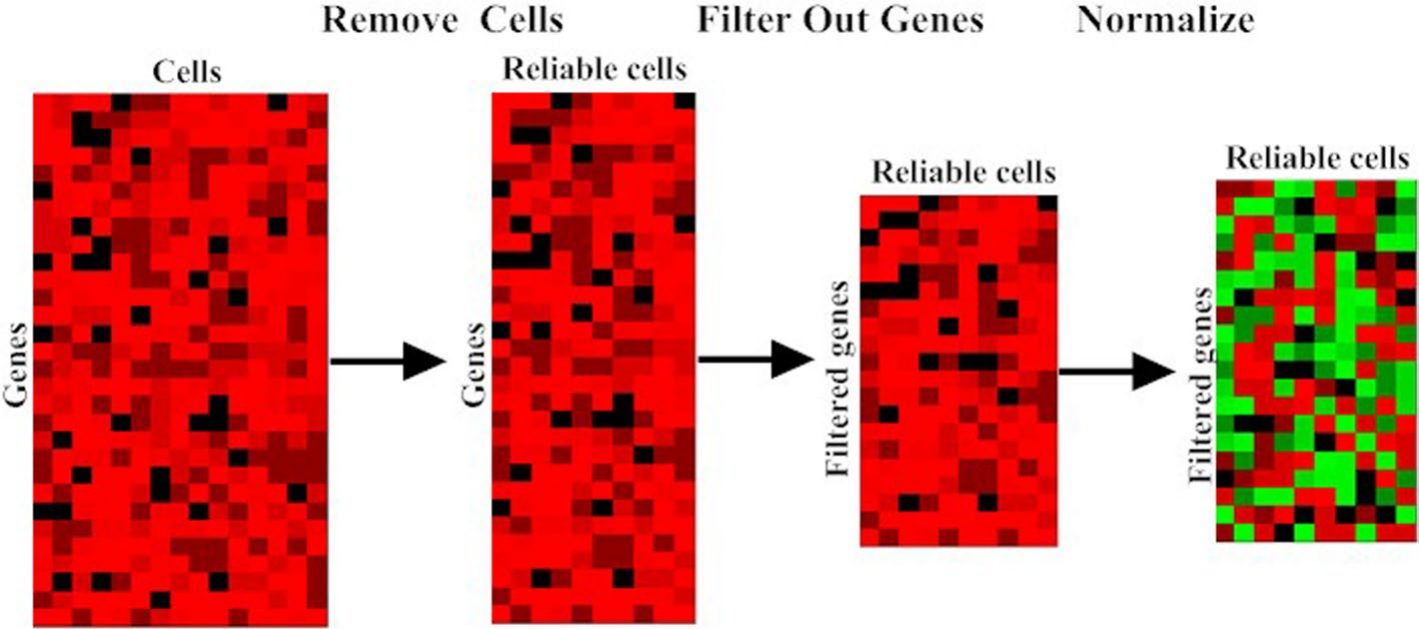
Single cell data preprocessing steps. Overview of the workflow for the data preprocessing. First, unreliable cells with low quality were removed. Then genes with low expression values were filtered out. Finally, the cleaned data set was normalized for downstream analysis

To eliminate noises or missing data that are contained in the dataset, a data preprocessing procedure is carried out first. As shown in Fig. 3, it consists of the following steps.

#### Step 1: removing cells with low sequencing quality

RNA-SeQC tool is used to remove cells with low-quality sequencing values [29], if the number of total reads is less than 3,000,00 or the mapping rate is less than 50$\%$ or the number of detected genes is less than 2000 or the portion of intergenic region is more than 30$\%$, the cells are identified as outlier cells, which are excluded for further analysis.

#### Step 2: filtering out genes with low expression values

First, genes with a transcript per million (TPM) value less than 1 are considered unreliable and replaced with 0; second, TPM values are log2-transformed after adding a value of 1 (log2(TMP+1)) in order to reduce the effect of highly expressed genes; and third, genes expressed in $<10\%$ of the bulk groups are discarded.

#### Step 3: normalizing gene expression data

For removing systematic variation in an experiment which affects the measured gene expression levels and examining relative expression levels, the gene expression data are first centered by subtracting the average expression of each gene from all cells, and then are divided by the variance of each gene from all cells.

### Improvement of spectral clustering

Let $P=\{p_1,p_2,\ldots ,p_n\}$ denote a given set of data points, where each data point $p_i$ is a *r* dimensional column vector, $S=(s_{ij})\in R^{n\times n}$ is a symmetric similarity matrix, where $s_{ij}\ge 0$ is a measure of the similarity between data points $p_i$ and $p_j$, a greater value of $s_{ij}$ indicates data points $p_i$ and $p_j$ are more similar. In conventional spectral clustering, we are trying our best to construct a *k* dimensional column feature vector $x_i$ for each data point $p_i$, where *k* is far less than *r*. Intuitively, if two data points are more similar, their feature vectors should be closer to each other in the feature space. Then each data point can be represented by a *k* dimensional feature vector. Therefore, the problem of finding *k* dimensional feature vectors can be converted into the following optimization problem:

1 $$\begin{aligned} &\underset{{{x}_{i}}\in {{R}^{k}},i=1,2,\ldots ,n}{\mathop {\text {minimize}}}\,\frac{1}{2}\sum \limits _{i,j=1}^{n}{{{s}_{ij}}||{{x}_{i}}-{{x}_{j}}|{{|}^{2}}}\\&\text {subject}\ \text {to}\ \sum \limits _{i=1}^{n}{{{x}_{i}}x_{i}^{T}={{I}_{k}}} \end{aligned}$$

where $I_k$ is a unit matrix. Let *D* be a diagonal matrix that has its *l*th diagonal entry equals to the sum of all elements in the *l*th row of the similarity matrix, then one can calculate the Laplacian matrix as $L=D-S$. Define a feature $n\times k$ matrix $M=[m_1,m_2,\ldots ,m_k]$, where $m_j$ is the unit eigenvector corresponding to the *j*th minimum eigenvalue of the Laplacian matrix *L*. Let $x_i$ be the *i*th row of matrix *M*. Then it can be proved that $x_i(i=1,2,\ldots ,n)$ is the solution of the above optimization problem (1). With these *k* dimensional features of all data points, any feature-based clustering method can be used to perform cluster analysis.

We improve the conventional spectral clustering by taking much account of the dissimilarities between data points. A symmetric dissimilarity matrix $DS=(ds_{ij})\in R^{n\times n}$ is used to define the dissimilarities between data points, where $ds_{ij}\le 0$, the smaller this value, the more dissimilar between data points $p_i$ and $p_j$. We are also trying to get a *k* dimensional column feature vector $y_i$ for each data point $p_i$, where *k* is far less than *r*. Analogously, if two data points are more dissimilar, their feature vectors should be more distant to each other in the feature space. So the optimization problem can be formulated as follows:

2 $$\begin{aligned} &\underset{{{y}_{i}}\in {{R}^{k}},i=1,2,\ldots ,n}{\mathop {\text {minimize}}}\,\frac{1}{2}\sum \limits _{i,j=1}^{n}{d{{s}_{ij}}||{{y}_{i}}-{{y}_{j}}|{{|}^{2}}}\\&\text {subject}\ \text {to}\sum \limits _{i=1}^{n}{{{y}_{i}}y_{i}^{T}={{I}_{k}}} \end{aligned}$$

Considering similar and dissimilar representation problems meanwhile, we are attempting to find a *k* dimensional column feature vector $z_i$ for each data point $p_i$, where *k* is far less than *r*. If two data points are more similar, their feature vectors should be closer to each other while if two data points are more dissimilar, their feature vectors should be more distant to each other in the feature space. Therefore, by some simple algebraic manipulation we can join optimization problem (1) and (2) to obtain the following equivalent expression:

3 $$\begin{aligned}&\underset{{{z}_{i}}\in {{R}^{k}},i=1,2,\ldots ,n}{\mathop {\text {minimize}}}\,\frac{1}{2}\sum \limits _{i,j=1}^{n}{[(1-\omega )\cdot {{s}_{ij}}+\omega \cdot d{{s}_{ij}})]||{{z}_{i}}-{{z}_{j}}|{{|}^{2}}} \\&\text {subject}\ \text {to}\sum \limits _{i=1}^{n}{{{z}_{i}}z_{i}^{T}={{I}_{k}}} \end{aligned}$$

where $0\le \omega \le 1$ is a parameter that is used to balance the similarity and dissimilarity described by feature vectors. Obviously, when $\omega =0$, problem (3) is transformed into optimization problem (1), while when $\omega =1$, problem (3) is transformed into optimization problem (2). $W=(1-\omega )S+\omega DS=(w_{ij})\in R^{n\times n}$ is a weighted symmetric incidence matrix that defines the relationship between data points, if $w_{ij}>0$ this indicates that the data points $p_i$ and $p_j$ are similar, if $w_{ij}<0$ this shows that the data points $p_i$ and $p_j$ are distant, if $\omega _{ij}=0$ this means that data points $p_i$ and $p_j$ are irrelevant. Let $D'=diag(d'_{11},d'_{11},\ldots ,d'_{nn})$ is a diagonal matrix with $d_{ii}^{\text { }\!\!'\!\!\text { }}=\underset{j=1}{\overset{n}{\mathop \sum }}\,{{w}_{ij}}$, a generalized Laplacian matrix $L'$ is defined as $L'=D'-W$. Let $z_i$ be the *i*th column of matrix *Z*. Then it can be proved that the problem (3) could be transformed into the following problem:

4 $$\begin{aligned}{}&\underset{{{z}_{i}}\in {{R}^{k}},i=1,2,\ldots ,n}{\mathop {\text {minimize}}}\,tr(Z{L}^{\prime}{{Z}^{T}}) \\ & \text {subject to}\ Z{{Z}^{T}}={{I}_{k}}\end{aligned}$$

where $Z=[z_1,z_2,\ldots ,z_n]\in R^{k\times n}$ and *tr* denotes the matrix trace. This is the standard form of a trace minimization problem. It can be proved that *Z* consists of the eigenvectors corresponding to the first *k* minimum eigenvalues of $L'$ is the solution to the problem (4). Then we can use any feature-based clustering algorithm on the first *k* eigenvectors to cluster data points.

### Identifying cell types using improved spectral clustering

**Fig. 4:**
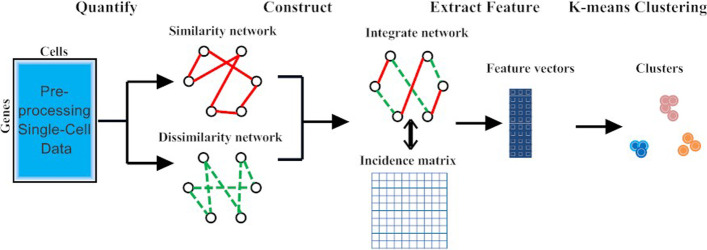
The framework of improved spectral clustering for grouping cells. Cell-cell similarity/dissimilarity networks were construct and then were integrated to build an incidence matrix. Selection features were used directly by K-means algorithms to assign cells to clusters

After preprocessing the single-cell dataset, constructing an appropriate incidence matrix is key to cluster single cells by improved spectral clustering. The detailed steps, depicted in Fig. 4, are given as follows.

#### Quantifying pairwise similarities and dissimilarities

Spearman’s rank correlation coefficient (denoted by the Greek letter $\rho$) is a non-parametric measure of correlation that assesses the relationship between two variables without making any assumptions, we use it to measure the similarity/dissimilarity between cells. The $\rho$ of two cells (*i* and *j*) is calculated as:

5 $$\begin{aligned} \rho (i,j)=1-\left( \frac{6\sum \limits _{t=1}^{m}{{{d}_{t}^{2}}}}{m({{m}^{2}}-1)}\right) \end{aligned}$$

where *m* is the number of genes, $d_t$ represents the difference between the two numbers in *t*th pair of gene ranks. It can vary between -1 and 1. The similarity *s*(*i*, *j*) and dissimilarity *ds*(*i*, *j*) between cell *i* and cell *j* can then be calculated as:

6 $$\begin{aligned}&s(i,j)=\left\{ \begin{aligned}&\rho (i,j)\quad if\,\rho (i,j)>0 \\&0\ \quad \quad \quad otherwise \\ \end{aligned} \right. , \\&ds(i,j)=\left\{ \begin{aligned}&\rho (i,j)\quad if\,\rho (i,j)<0 \\&0\ \quad \quad \quad otherwise \\ \end{aligned} \right. \end{aligned}$$

If the $\rho$ between cell *i* and cell *j* is close to 1, which represents the gene expression levels of cell *i* and cell *j* tend to be relatively high or low simultaneously, in other words, cell *i* and cell *j* have semblable gene expression patterns, the higher the $\rho$ is, the greater the similarity is. Likewise, if the $\rho$ between cell *i* and cell *j* is close to -1, which means the gene expression levels of cell *i* and cell *j* appear to have an opposite trend, that is to say, there is a large dissimilarity between the gene expression patterns of cell *i* and cell *j*, the lower the $\rho$ is, the stronger the dissimilarity is. Besides Spearman’s rank correlation coefficient, Pearson’s correlation coefficient can be used to calculate the similarity/dissimilarity among cells.

#### Constructing incidence matrix based on similarities and dissimilarities

For each cell *i*, the similarities between cell *i* and every other cell are sorted in descending order, and the dissimilarities between cell *i* and every other cell are sorted in ascending fashion. The similarity matrix $S=(s_{ij})\in R^{n\times n}$ is designed as follows: for cell *i* and cell *j*, if cell *i* is among the top *h* similar cells of cell *j*, or cell *j* is among the top *h* similar cells of cell *i*, then $s_{ij}=s_{ji}=s(i,j)=s(j,i)$; otherwise, $s_{ij}=s_{ji}=0$. Likewise, the dissimilarity matrix $DS=(ds_{ij})\in R^{n\times n}$ is built as follows: for cell *i* and cell *j*, $ds_{ij}=ds_{ji}=ds(i,j)=ds(j,i)$ if *ds*(*i*, *j*) is in the top *q* of the sorted dissimilarity list of cell *i* or *ds*(*j*, *i*) is in the top *q* of the sorted dissimilarity list of cell *j* ; otherwise, $ds_{ij}=ds_{ji}=0$.

The incidence matrix *W* is constructed by incorporating similarity matrix *S* with dissimilarity matrix *DS* using the following equation:

7 $$\begin{aligned} W=(1-\omega )S+\omega DS \end{aligned}$$

where $\omega$ is selected from the set $\{0, 0.1, 0.2, 0.3, 0.4, 0.5, 0.6, 0.7, 0.8, 0.9, 1\}$, $\omega$ is used to trade off the proportion of similarity and dissimilarity in the incidence matrix.

#### Extracting feature vectors for K-means clustering

After constructing a incidence matrix *W* by the way described above, we can get a generalized Laplacian matrix $L'=D'-W$, where $D'$ is a diagonal matrix with the row-sums of *W* on the diagonal and zeros in the off-diagonal elements. If the number of clusters is *k*, the first *k* eigenvectors $u_{1},u_{1},\ldots ,u_{k}$ of the generalized Laplacian matrix $L'$ are calculated. Let $u_{1},u_{1},\ldots ,u_{k}$ be the columns of matrix $U\in R^{n\times k}$, the *i*th row of *U* would be the feature vector corresponding to cell *i*. Then k-means algorithm is performed to cluster cells with these feature vectors by using MATLAB’s kmeans function.

### Evaluation metrics

In this study, four indices are employed to evaluate the performance of improved spectral clustering and conventional spectral clustering algorithm, including Purity, RI, ARI and NMI. Let the $C_U$-partition $U=\{U_1,U_2,\ldots ,U_{C_U}\}$ be our calculation partition of n data points $p_1$, $p_2$, $\cdots$, $p_n$, the $C_V$-partition $V=\{V_1,V_2,\ldots ,V_{C_V}\}$ be the genuine partition. We can define the contingency table $T=(t_{ij})\in R^{C_U\times C_V}$, where entry $t_{ij}$ is the number of data points that are both in cluster $U_i$ and $V_j$. Each obtained cluster $U_i(i=1,2,\ldots ,C_U)$ is assigned to the cluster $V_j(j=1,2,\ldots ,C_V)$ which has the largest number in the *i*th row of contingency table, and then the accuracy of this assignment is computed by the sum of the entry of the best assigned in the contingency table by the total number of data points (N):

8 $$\begin{aligned} Purity(U,V)=\frac{1}{N}\sum \limits _{i=1}^{{{C}_{U}}}{\max }({{t}_{i.}}) \end{aligned}$$

where $t_i.$ denotes the elements in the *i*th row of contingency table, *max*() is the largest element.

RI measures the fraction of pairs of data points that are classified in the same way in both clusterings with the number of pairs of all data points. Thus, it is defined by:

9 $$\begin{aligned} RI(U,V)=\frac{2({{n}_{00}}+{{n}_{11}})}{N(N-1)} \end{aligned}$$

where $n_{00}$ denotes the size of pairs that are in different clusters under *U* and *V*, $n_{11}$ denotes the size of pairs that are in the same cluster under *U* and *V*.

ARI is the normalized difference of the RI and its expected value under the assumption that a generalized hypergeometric distribution as null hypothesis [30]. Mathematically, it is defined as follows:

10 $$\begin{aligned} ARI(U,V)=\frac{\sum \nolimits _{i=1}^{{{C}_{U}}}{\sum \nolimits _{j=1}^{{{C}_{V}}}{\left( \begin{aligned}&{{t}_{ij}} \\&2 \\ \end{aligned} \right) -\frac{2}{N(N-1)}\sum \nolimits _{i=1}^{{{C}_{U}}}{\left( \begin{aligned}&{{t}_{i.}} \\&2 \\ \end{aligned} \right) \sum \nolimits _{j=1}^{{{C}_{V}}}{\left( \begin{aligned}&{{t}_{.j}} \\&2 \\ \end{aligned} \right) }}}}}{\frac{1}{2}\left[ \sum \nolimits _{i=1}^{{{C}_{U}}}{\left( \begin{aligned}&{{t}_{i.}} \\&2 \\ \end{aligned} \right) }+\sum \nolimits _{j=1}^{{{C}_{V}}}{\left( \begin{aligned}&{{t}_{.j}} \\&2 \\ \end{aligned} \right) } \right] -\frac{2}{N(N-1)}\sum \nolimits _{i=1}^{{{C}_{U}}}{\left( \begin{aligned}&{{t}_{i.}} \\&2 \\ \end{aligned} \right) \sum \nolimits _{j=1}^{{{C}_{V}}}{\left( \begin{aligned}&{{t}_{.j}} \\&2 \\ \end{aligned} \right) }}} \end{aligned}$$

where $t_{i.}=\sum \nolimits _{j=1}^{C_V}{t_{ij}}$ is the sum of row *i* in the contingency table *T*, $t_{.j}=\sum \nolimits _{i=1}^{C_U}{t_{ij}}$ is the sum of column *j* in the contingency table *T*. The ARI ranges from − 1 to 1. the larger ARI, the better the quality of clustering.

NMI provides a sound normalized indication to the comparison of clusterings, which has its origin in information theory and is based on the notion of entropy [31], it is defined as:

11 $$\begin{aligned} NMI(U,V)=\frac{\sum \nolimits _{i=1}^{{{C}_{V}}}{\sum \nolimits _{j=1}^{{{C}_{U}}}{{{t}_{ij}}}\log \frac{N\cdot {{t}_{ij}}}{{{t}_{i.}}\cdot {{t}_{.j}}}}}{\max (-\sum \nolimits _{i=1}^{{{C}_{V}}}{{{t}_{i.}}\cdot \log \frac{{{t}_{i.}}}{N},}-\sum \nolimits _{j=1}^{{{C}_{U}}}{{{t}_{.j}}\log \frac{{{t}_{.j}}}{N}})} \end{aligned}$$

where the numerator represents the mutual information between *V* and *U*, and the denominator denotes the entropy of the clusterings *V* and *U*.

We use these external indices to evaluate the agreement between the results of improved spectral clustering and the true clusters, and the agreement between the results of conventional spectral clustering and the true clusters, respectively. The more the agreement, the better the performance of the clustering method.

## Data Availability

Some datasets supporting the conclusions of this article are available in the GEO database repository under accession numbers GSE75688, others are from previously published papers [23] and [24], and are freely available at https://github.com/BatzoglouLabSU/SIMLR and https://github.com/ishspsy/project/tree/master/MPSSC/Data. The Matlab codes for our ISC method ia available at https://github.com/Liyuanyuan1980/ISC

## Abbreviations

ARI: Adjusted Rand Index
BCCs: Breast cancer cells
CALISTA: Clustering and lineage inference in single cell transcriptional analysis
DCs: Dendritic cells
ESCs: Embryonic stem cells
ISC: Improved spectral clustering
MCs: A mixture of diverse single cells
NCs: Neuronal cells
NMF: Nonnegative matrix factorization
NMI: Normalized mutual information
PCA: principal component analysis
PSSC: Pairwise sparse spectral clustering
RI: Rand Index
SC: Spectral clustering
SC3: Single-cell consensus clustering
SNN: Shared nearest neighbor
SSC: Sparse spectral clustering
TPM: Transcript per million
